# Mouse Yolk Sac Hematopoiesis

**DOI:** 10.3389/fcell.2018.00080

**Published:** 2018-07-20

**Authors:** Toshiyuki Yamane

**Affiliations:** Department of Stem Cell and Developmental Biology, Mie University Graduate School of Medicine, Tsu, Japan

**Keywords:** yolk sac, blood cell development, embryonic hematopoiesis, fetal hematopoiesis, B-1 B cell, macrophage

## Abstract

The yolk sac is the first observed site of hematopoiesis during mouse ontogeny. Primitive erythroid cells are the most well-recognized cell lineages produced from this tissue. In addition to primitive erythroid cells, several types of hematopoietic cells are present, including multipotent hematopoietic progenitors. Yolk sac-derived blood cells constitute a transient wave of embryonic and fetal hematopoiesis. However, recent studies have demonstrated that some macrophage and B cell lineages derived from the early yolk sac may persist to adulthood. This review discusses the cellular basis of mouse yolk sac hematopoiesis and its contributions to embryonic and adult hematopoietic systems.

## Anatomy and phylogeny

**Figure 1:**
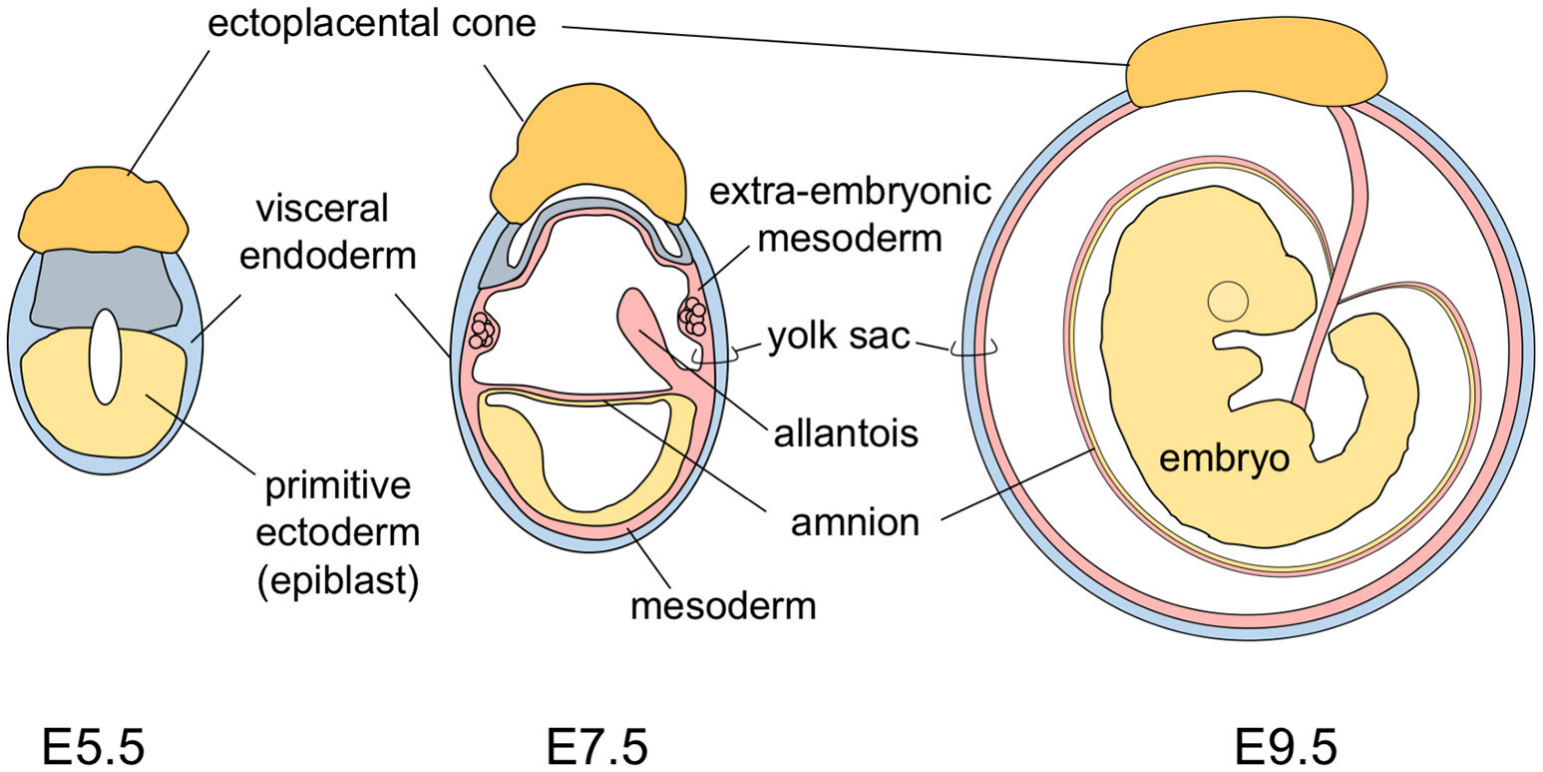
A schematic representation of mouse development from stages E5.5 to E9.5.

The yolk sac tissue consists of two layers: a visceral endoderm layer derived from the primitive endoderm (hypoblasts) and an extra-embryonic mesoderm layer derived from epiblast cells that make an ingress through the primitive streak (Figure 1). In the early developmental stage, the mouse embryo is U-shaped, with the neuroectoderm inside and the gut endoderm outside. As development progresses, the turning of mouse embryos eventually reverses this topology to the observed standard positioning of vertebrate embryos. This movement results in mouse embryos being completely surrounded by the amnion and the yolk sac (Figure 1) (Kaufman and Bard, 1999). This anatomically distinguishes the mouse yolk sac from those of other vertebrates, including humans in which the yolk sac is attached to, but does not envelop, the embryo.

The yolk sac is the first site of hematopoiesis in mammalian embryos. In phylogenetic studies, yolk sac blood islands have been observed in some ray-finned fish species (Al-Adhami and Kunz, 1976; Iuchi and Yamamoto, 1983); however, the zebrafish, a key ray-finned fish laboratory model, produces blood cells not in the yolk sac but intra-embryonically in a site known as the intermediate cell mass of Oellacher (Al-Adhami and Kunz, 1977). In some amphibian embryos, especially those with large yolks, the bulk of the yolk is not incorporated into the gut, and a yolk sac structure is formed. However, in amphibian embryos with small amounts of yolk, the yolk is incorporated into the gut during early development, with no formation of a yolk sac structure (Duellman and Trueb, 1994). In this type of amphibian embryo, blood cells are initially formed ventrally, at sites known as ventral blood islands, which are considered to be a counterpart of yolk sac hematopoiesis (Turpen et al., 1997). As in mammals and some ray-finned fish species, avian embryos produce blood cells in the yolk sac. There are few studies in the literature regarding non-avian reptilian embryonic hematopoiesis; however, in the turtle, yolk sac blood islands are known to be the first sites of hematopoiesis (Vasse and Beaupain, 1981).

## Primitive erythropoiesis

Hematopoiesis is observed in mouse embryos as early as embryonic day 7.5 (E7.5) in the extra-embryonic mesoderm layer of the yolk sac. Hematopoietic cells are observed as cell aggregates, termed blood islands, until yolk sac vasculatures are firmly formed. Primitive erythroid cells constitute the primary cell lineage produced in yolk sac blood islands, and the yolk sac is the exclusive site of primitive erythropoiesis. Primitive erythroid cells have hemoglobin proteins different from those of late fetal and adult erythroid lineage cells (definitive erythroid cells) (Zon, 1995). In mice, primitive erythroid cells express embryo-specific β globins (ε_y_, β_H1_) and an α globin (ζ), but only low levels of adult β globins (β_major_, β_minor_) and α globins (α_1_, α_2_) (Whitelaw et al., 1990; Palis et al., 2010). These embryo-specific globins have a higher affinity for oxygen than do adult globins (Wells and Brittain, 1981), which presumably enhances the maternal–embryonic gas exchange in the placenta. The earliest movement of primitive erythroid cells into embryonic circulation is observed when embryos possess 5 or 6 somite pairs (E8), coinciding with heartbeat onset (Ji et al., 2003). Initially, a nucleated form of primitive erythroid cells circulates in the embryonic blood stream. Eventually, these primitive erythroid cells enucleate between E12.5 and E16.5 (Kingsley et al., 2004). Mouse embryos lacking primitive erythropoiesis do not develop beyond E11.5 (Fujiwara et al., 1996; Tsang et al., 1998). In contrast, mouse embryos lacking definitive (late fetal) hematopoiesis have been known to survive to E15 (Mucenski et al., 1991). Therefore, primitive erythroid cell-mediated oxygen supply to embryonic tissues appears to be essential for mid-gestational mouse embryos.

## Megakaryocyte and macrophage lineages

In addition to the large cell population of erythroid cells, cells expressing megakaryocyte lineage markers have been observed in the E9.5 yolk sac (Tober et al., 2007). Interestingly, diploid cells form long extensions of cytoplasm, known as proplatelets, in the E10.5 yolk sac for the release of platelets (Potts et al., 2014). Platelets derived from these cells appear to be released into the blood stream at approximately E11, and these yolk sac-derived platelets are larger in size compared to adult bone marrow-derived platelets (Tober et al., 2007). These yolk sac cells are likely to be the initial source of platelets in mouse embryos rather than the polyploid megakaryocytes that form later in the fetal liver. Immature macrophages have also been observed in the E9 yolk sac by morphological analysis, and it was reported that these macrophages have a proliferative capability *ex vivo* (Takahashi et al., 1989). Macrophage colony-forming capability of the yolk sac has been observed as early as E7.0 in parallel with the appearance of primitive erythroid colony-forming cells (Palis et al., 1999). Furthermore, cells which highly express the CX_3_CR1 knock-in reporter, a monocyte/macrophage marker, have been observed in the E10 yolk sac (Bertrand et al., 2005).

## Multipotent hematopoietic progenitor cells

The ability of yolk sac cells to generate blood cell lineages is not restricted to primitive erythroid cells, platelets, and macrophages. Earlier studies using *in vitro* colony formation assays have revealed the presence of definitive (late fetal and adult) erythroid progenitors, granulocyte/macrophage progenitors, and common progenitors for erythro-myeloid lineages in the yolk sac, especially after E9 (Palis et al., 1999; Ferkowicz et al., 2003). These yolk sac progenitors are referred to as erythroid–myeloid progenitors (EMPs). Lymphoid lineage potentials are hallmarks of multipotent hematopoietic progenitor cells. Although lymphoid lineage potentials generally cannot be examined in colony assays, with the exception of B cell lineage-committed progenitors that form small colonies in the presence of IL-7 (Hayashi et al., 1990; Yamane et al., 2001), co-culturing with stromal cell lines or transplantation into mice has revealed the presence of lymphoid lineage potentials in the yolk sac.

Co-culturing with stromal cell lines has shown that the early yolk sac cells at E7.5–E8.5 are not sufficiently potent to give rise to lymphocytes (Yokota et al., 2006). Flow cytometry analysis at E8.5 has revealed only a small number of cells positive for CD45, a non-erythroid pan-blood cell marker (Yamane et al., 2013). In contrast, yolk sac cells isolated at ~ E9.5, when the CD45^+^ cell population is increased, displayed a high potency to generate T and B cells (Yamane et al., 2009). Weissman et al. (1978) demonstrated that E8 and E9 yolk sac cells transplanted *in utero* into the yolk sac cavities of same-aged hosts gave rise to T cells. E9.5 yolk sac-derived T progenitors gave rise to both αβ and γδ T cell lineages in an unbiased manner (Yamane et al., 2009; Yoshimoto et al., 2012). This is in contrast to yolk sac-derived B progenitors, which preferentially differentiate into the B-1 B cell lineage (discussed below). However, it is unknown if the yolk sac-derived T cell progenitors have non-biased Vγ gene usage. This intriguing question remains unanswered because T cells have different Vγ gene usage patterns in different tissues, and some γδ T cell subsets are solely derived from the fetal stage (Havran and Allison, 1988; Ikuta et al., 1990; Haas et al., 2012).

Hematopoietic cells in E9.5 yolk sacs express very few, if any, IL-7 receptors, which are expressed by lymphoid-restricted progenitors (Böiers et al., 2013). Additionally, E9 and E10 yolk sacs have only minimal *Rag-1* reporter expression compared to fetal liver hematopoietic cells (Yokota et al., 2006; Böiers et al., 2013). Therefore, it is likely that the yolk sac is not the primary site of lymphoid differentiation. Rather, the yolk sacs bear multipotent hematopoietic cells with lymphoid lineage potentials. Cells with the CD45^+^Kit^high^AA4.1^+^ phenotype in the E9.5 yolk sac, which account for approximately 5% of CD45^+^ yolk sac cells and show differentiation potency for multilineage cells, including erythroid–myeloid and lymphoid lineage cells, can explain the lymphoid potentials of the yolk sac (Yamane et al., 2009; Ito et al., 2013). Similarly, a recent report showed that exclusion of CD11a-positive cells may further enrich the multipotent hematopoietic progenitor fraction with lymphoid potentials in the E9.5 yolk sac (Inlay et al., 2014).

## Hematopoietic stem cells

Despite the presence of multipotent cells, early yolk sac hematopoietic cells (up to E9.5) lack hematopoietic stem cell (HSC) long-term repopulation activity (Yamane et al., 2013). Embryonic portions, as well as the extra-embryonic yolk sac, lack HSC activity in the early developmental stages (Cumano et al., 1996; Arora et al., 2014). HSCs with long-term repopulation ability appear at E10.5–11.5 in multiple locations, including the para-aortic region (Medvinsky and Dzierzak, 1996), vitelline and umbilical arteries (de Bruijn et al., 2000), yolk sac (Huang and Auerbach, 1993), placenta (Gekas et al., 2005; Ottersbach and Dzierzak, 2005), and head region (Li et al., 2012). Collectively, these studies suggest that the appearance of multipotent erythroid–myeloid and lymphoid potentials precedes the appearance of post-natal long-term repopulation HSC activity, especially in the yolk sac.

Whether hematopoietic cells in the early yolk sac give rise to HSCs in the late yolk sac and body or not is a controversial topic. Labeling early yolk sac cells by activating *Rosa* reporters using tamoxifen in *Runx1-MerCreMer* mice resulted in the detection of *Rosa* reporters in multiple adult blood cell lineages (Samokhvalov et al., 2007). However, detection was limited to only a small fraction of adult blood cells, with the exception of microglia and other macrophages (discussed below) (Ginhoux et al., 2010). Given that there were trace amounts of administered tamoxifen, discerning minimal reporter expression from possible reporter activation of late hematopoietic cells in other embryonic locations was difficult. In addition, a report showed that haploinsufficiency of *Runx1* has a promotive effect on the transplantability of E10-11 yolk sac hematopoietic cells (Cai et al., 2000). These experimental approaches of the study make it hard to draw a conclusion regarding the fate of early yolk sac hematopoietic cells.

**Figure 2:**
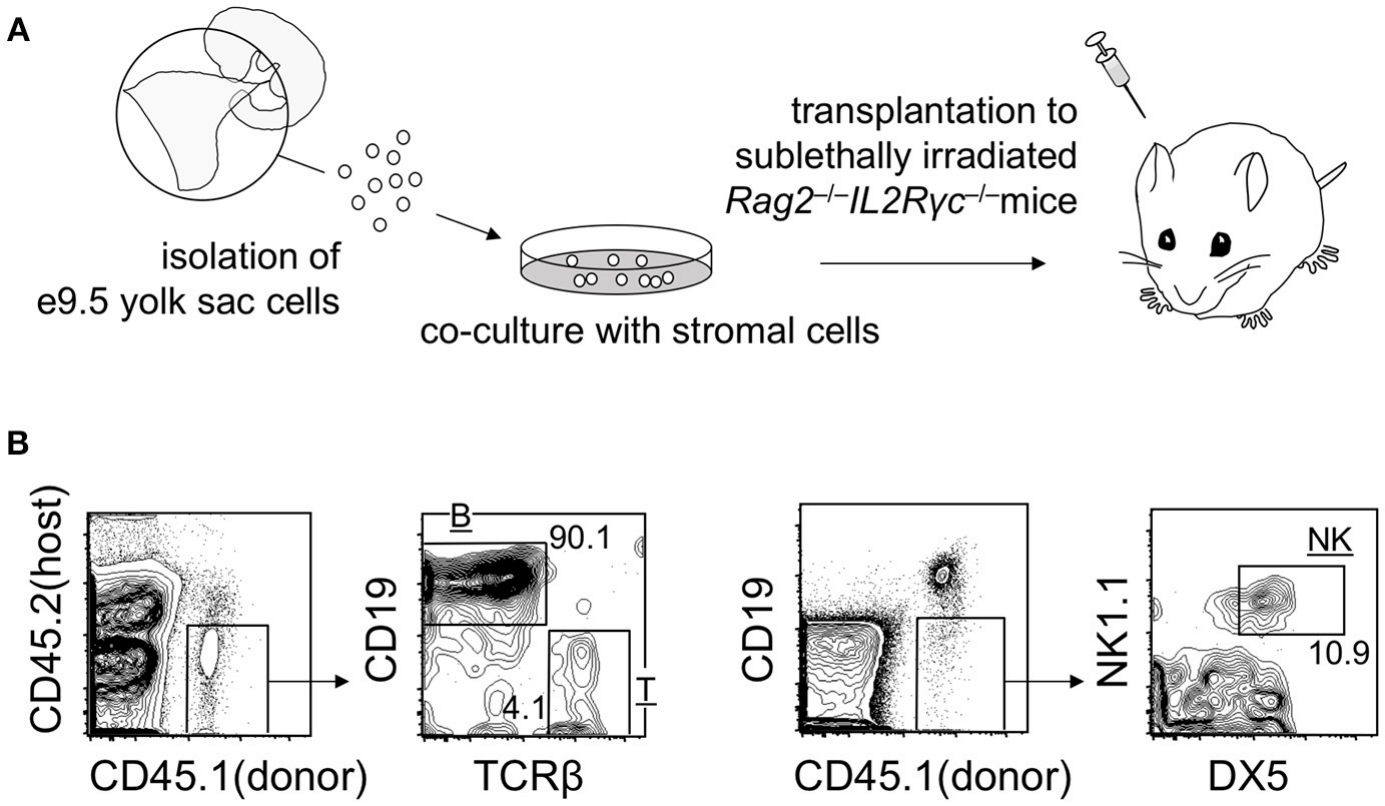
Repopulation of lymphoid lineage cells by E9 yolk sac-derived cells. **(A)** Overview of the experimental procedure. **(B)** Representative flow cytometry plots showing T, B, and NK donor cell populations in the spleen at 3 weeks post-transplantation.

As mentioned above, transplantation of early yolk sac cells (up to E9.5) into post-natal mice does not lead to the engraftment of cells. However, it is noteworthy that E9.5 yolk sac cells cultured on stromal cells for 1–2 weeks engrafted in adult mice repopulate T, B, and natural killer (NK) lymphoid compartments, but not myeloid cell lineages. B cell lineage repopulation was particularly more efficient than that of T and NK cell lineages (Figure 2) (Ito et al., 2013). These observations suggest that engraftment failure of early yolk sac cells originates at the multipotent progenitor level, not at the committed progenitor level. Although T and NK cells and conventional follicular B cells are replaced by HSC-derived cells, B-1 B cells establish a self-replenishable pool in the body cavity. Once established, the pool is not replaced by adult HSCs (Lalor et al., 1989). Therefore, even if the early yolk sac cells do not contribute to HSC compartment, the B-1 B cell pool can be established and maintained via early yolk sac-derived cells. Recent reports have suggested that early yolk sac-derived macrophage lineages may also persist in the body for a long time. These early yolk sac cell contributions are discussed below.

## Colonization of the fetal liver

At E10–11, hematopoietic cells colonize the hepatic primordia (Houssaint, 1981; Zovein et al., 2010). The vast majority of cells that initially seed the fetal liver are likely to be yolk sac-originated cells. This is because yolk sac hematopoiesis precedes that of the aortic and placental region, and the yolk sac contains the largest pool of blood cells at this stage (Lux et al., 2008). Additionally, the vitelline vein from the yolk sac drains directly into the fetal liver (Kaufman, 1992; Zovein et al., 2010). Therefore, it is likely that early yolk sac cells establish the first wave of hematopoiesis in the fetal liver. Most of the hematopoietic cells, except for primitive erythroid cells, have a CD45^+^KIT^+^ cell surface phenotype at this stage of fetal liver development. ~10% of the cell population expresses AA4.1 on the cell surface. In contrast, expression of Sca-1, an HSC marker, is observed in <1% of the cell population at this stage. While Sca-1 is likely expressed in aortic region-derived hematopoietic cells (Miles et al., 1997), CD45^+^KIT^+^AA4.1^+^ E9.5 yolk sac multipotent hematopoietic progenitor cells do not express Sca-1 (Yamane et al., 2009). Taking these observations into account, early yolk sac-derived multipotent hematopoietic cells are likely to be the major hematopoietic source in the very early stage of fetal liver hematopoiesis. Lymphomyeloid progenitors that lack erythroid and megakaryocytic potentials have been observed in fetal liver at E11.5, and have CD45^+^KIT^+^ FLT3^+^IL7Rα^+^ cell surface phenotypes (Böiers et al., 2013). It is not known if these lymphomyeloid progenitors are a contiguous cell population from the multipotent hematopoietic progenitor cells in the yolk sac. Examination of E10.5 fetal liver will help to determine the origin of these cells.

## Persistence of yolk sac-originated cells to adulthood

### B-1 B cells

As mentioned above, early yolk sac cells generate the B lymphocyte lineage. However, early yolk sac-derived B cell progenitor cells have a strong tendency for differentiation into B-1 B lymphocytes (Yoshimoto et al., 2011; Ito et al., 2013), whereas differentiation into follicular B cells and splenic marginal zone B cells (B-2 B cell lineages) is minimal (Ito et al., 2013). This tendency is not restricted to yolk sac-derived B progenitor cells, but is also observed in cells derived from the caudal halves of the embryo (Ito et al., 2013), or specifically the para-aortic splanchnopleura-derived B progenitor cells (Godin et al., 1993). These findings suggest that this is a common feature in hematopoietic cells appearing during the early developmental stages. Experimentally, early yolk sac-derived B progenitor cells can be engrafted in adult mice and permanently self-replenish in recipient mice (Ito et al., 2013). It is known that the B-1 B cell pool is established during the fetal period (Lalor et al., 1989). However, the physiologically relative contributions of yolk sac multipotent hematopoietic progenitor cells and fetal liver HSCs are unknown and warrant further study.

Follicular B cells produce antibodies mainly against protein antigens, with the help of T cells for activation. However, B-1 B cells produce antibodies against polysaccharides and phospholipids in a T cell-independent manner. Although the biological importance and relevance of the differentiation tendency of B progenitor cells in the early developmental stage is unknown, initial antibody production may be specialized to the B-1 IgM repertoire during development, given that a broad antibody repertoire derived from follicular B cells is obtained by the fetus via maternal IgG passed through the placenta.

### Macrophage lineage

The early yolk sac origin of adult microglial cells has been revealed (Ginhoux et al., 2010). In this study, tamoxifen administration at E7.0–E7.5 efficiently labeled >30% of microglial cells in the brain using *Runx1-MerCreMer/Rosa* reporter mice as aforementioned, whereas there was only limited fluorescent labeling of adult peripheral blood lineages (Ginhoux et al., 2010). Initial seeding of macrophages in various tissues during the mid-gestational stage is likely attributed to yolk sac-originated hematopoietic cells (Schulz et al., 2012). Persistence of yolk sac-derived macrophage lineages in adult tissues is prominent in microglial cells in the brain. Tissue-resident macrophages in the liver, lung, and epidermis may also be from early yolk sac-derived cells, yet they are limited in comparison to microglial lineage cells (Perdiguero et al., 2015). Because the yolk sac-derived macrophage progenitor cells reside in the brain before the establishment of the blood–brain barrier (Ginhoux et al., 2010), the immunologically privileged state of the brain after blood–brain barrier establishment may hamper further recruitment of microglial progenitor cells (Ajami et al., 2007; Zhao et al., 2015).

## Identification of diverse types of hematopoietic cells in the yolk sac

The lineage relationship of hematopoietic cells observed in the yolk sac has not been well-characterized compared with adult bone marrow hematopoietic cell compartments. The hematopoietic progenitors with erythromyeloid potential in the yolk sac are referred to as EMP. This term was initially used for the colony-forming cells with an erythromyeloid phenotype in the yolk sac (Bertrand et al., 2005). However, the term has been used differently by various groups, leading to its ambiguous definition. To avoid confusion, we use the term EMP to refer to the cells that give rise to definitive erythroid cells and the granulocyte/macrophage lineage, but not to primitive erythroid cells and lymphoid cells. Although CD41 (and KIT) are often used to identify EMPs in the early yolk sac (Chen et al., 2011; Hoeffel et al., 2015), the cell fractions separated by these two markers is still heterogenous (as discussed below), and the early yolk sac is likely to contain more variations of hematopoietic progenitors than previously thought.

The colony-formation assay revealed that the appearance of primitive erythroid precursors and mono-potent macrophage precursors precedes that of definitive erythroid and granulocytes/macrophages, and erythro-myeloid precursors in the yolk sac (Palis et al., 1999). The former colonies have been observed as early as E7.0, whereas the latter EMP-type colonies were observed mainly after E9 (Palis et al., 1999; Ferkowicz et al., 2003). Cells expressing mid-levels of CD41 were reported to have generated only primitive erythroid colonies and mono-potent macrophage colonies in the E7–E8 yolk sac, whereas CD41^high^ cells in the yolk sac observed after E8.25 were reported to preferentially give rise to EMP-type colonies (Ferkowicz et al., 2003). Additionally, it was shown that KIT^low^ cells, rather than KIT^high^ cells, are a preferential source of primitive erythroid and mono-potent macrophage colonies in the E8.5 yolk sac (McGrath et al., 2015).

It was reported recently that a cell subset expressing FcγR II (CD32) and/or FcγR III (CD16) within the CD41^+^KIT^high^ cell fraction present in the E8.5–9.5 yolk sac showed EMP readouts, but did not give rise to primitive erythroid cells. In addition, these FcγR II/III^+^CD41^+^KIT^high^ cells were reported to lack B cell lineage potential by stromal cell culture, whereas B cell potential was detected in non-FcγR II/III^+^CD41^+^KIT^high^ cell compartments of the E9.5 yolk sac (McGrath et al., 2015). Collectively, these properties of the FcγR II/III^+^CD41^+^KIT^high^ cell subset in the yolk sac highly resemble those of presumptive EMPs.

The cell fractionation method using distinct cell surface markers revealed that CD45^+^KIT^high^AA4.1^+^ cells contained within the CD41^+^ cell fraction in the E9.5 yolk sac show multipotent hematopoietic cell activity with definitive erythroid, myeloid, and lymphoid potentials, but are devoid of primitive erythroid potency (Yamane et al., 2009). Similarly, it was also reported that CD43^+^KIT^high^Sca1^+^CD11A^−^ cells in the E9.5–11.5 yolk sac, which is contained in the CD41^+^ cell fraction at least until E9.5, can give rise to erythromyeloid and lymphoid cells, although the cell population size seems very small (Inlay et al., 2014). Contrary to these reports, absolute lymphopoietic capability was observed in VE-cadherin^+^ cells within the CD41^−^ cell fraction (Yoshimoto et al., 2011, 2012).

**Figure 3:**
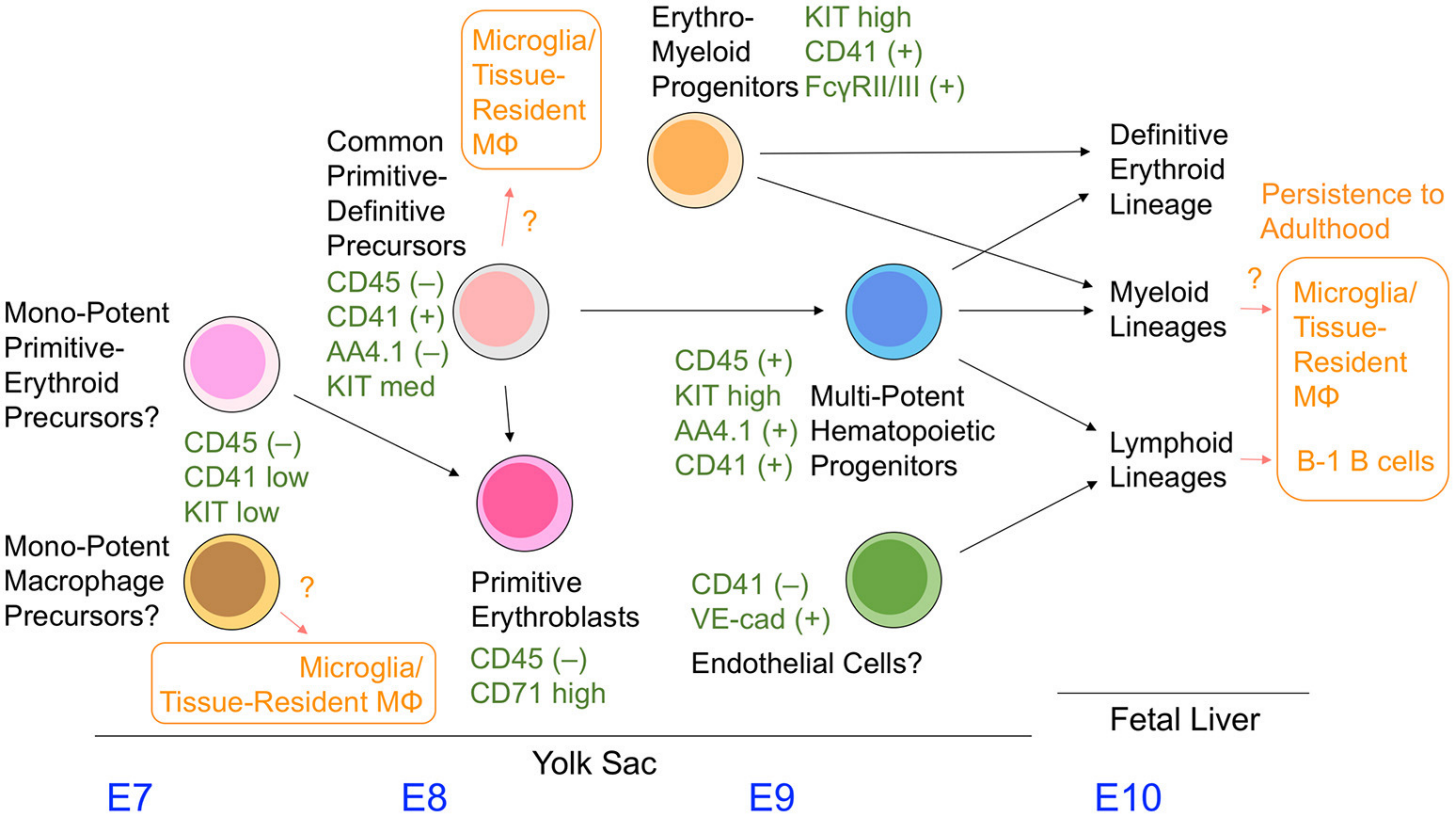
A model showing our current understanding of yolk sac hematopoiesis.

In addition, the more immature committed hematopoietic cells in the E8.5–9.5 yolk sac were identified as having a CD45^−^CD41^+^AA4.1^−^KIT^med^ cell surface phenotype. These cells give rise to the primitive erythroid lineage and CD45^+^KIT^high^AA4.1^+^ multipotent hematopoietic cell fraction (Yamane et al., 2013, 2017) (Figure 3); the presence of these cells has been previously observed in embryonic stem cell culture models (Kennedy et al., 1997; Perlingeiro et al., 2001). Even at a clonal level, CD45^−^CD41^+^AA4.1^−^KIT^med^ cells in the yolk sac can give rise to rearranged B lineage cells and embryo-specific β globin-expressing primitive erythroid cells simultaneously *in vitro* (Yamane et al., 2013). Therefore, the cell fraction present in the E8.5–9.5 yolk sac appears to serve as a common precursor for primitive erythroid cells and multipotent hematopoietic progenitors. Mechanistically, CD45^−^CD41^+^AA4.1^−^KIT^med−^ common precursor cells have a relatively high level of erythroid transcription factors, and can immediately give rise to CD45^−^CD71^high^ primitive erythroblasts (Yamane et al., 2017). Downregulation of erythroid transcription factor expression accompanies differentiation toward CD45^+^KIT^high^AA4.1^+^ multipotent hematopoietic progenitor cells, which is partly due to the action of the transcription factor Pu.1 (Yamane et al., 2017).

CSF1R (FMS)-expressing cells have been reported as early as E9 in mouse yolk sacs using the *MerCreMer* mouse transgenic line under control of the *Csf1r* promoter (Perdiguero et al., 2015). These cells are thought to give rise to microglial and first-wave embryonic macrophages. The above-mentioned multiple cell populations have a potency to differentiate into macrophage lineages. It remains unknown which cell population physiologically supplies these macrophage lineages (Figure 3). Additional markers such as FcγR II/III, CD45, AA4.1, and CD11A need to be examined together with CD41 and KIT to precisely characterize the hematopoietic cell populations in the yolk sac. In addition, the overlapping and hierarchical relationships between each cell population remain largely unknown and require further study. Figure 3 summarizes the information discussed in this review.

## Concluding remarks

Our understanding of mouse yolk sac hematopoiesis has greatly increased over the past few years. However, further studies are warranted to highlight the precise contributions of yolk sac hematopoietic cells to adult-stage hematopoietic cells. In addition, further research is needed to determine if yolk sac-derived macrophages are replaced when mice are exposed to infectious microbes (Machiels et al., 2017), given that most studies specifically use pathogen-free mice. Furthermore, it is critical to illustrate if the knowledge obtained to date is applicable to not only other animal models, but also organisms with longer lifespans, including humans. As technology and methodologies improve, our means to elucidate yolk sac hematopoiesis will be enhanced.

## Author contributions

The author confirms being the sole contributor of this work and approved it for publication.

### Conflict of interest statement

The author declares that the research was conducted in the absence of any commercial or financial relationships that could be construed as a potential conflict of interest.
